# Computational modelling of the long-term effects of brain stimulation on the local and global structural connectivity of epileptic patients

**DOI:** 10.1371/journal.pone.0221380

**Published:** 2020-02-06

**Authors:** Emmanouil Giannakakis, Frances Hutchings, Christoforos A. Papasavvas, Cheol E. Han, Bernd Weber, Chencheng Zhang, Marcus Kaiser

**Affiliations:** 1 Interdisciplinary Computing and Complex BioSystems (ICOS) Research Group, School of Computing, Newcastle University, Newcastle upon Tyne, England, United Kingdom; 2 Department of Electronics and Information Engineering, Korea University, Sejong, Republic of Korea; 3 Institute of Experimental Epileptology and Cognition Research, University of Bonn, Bonn, Germany; 4 Department of Functional Neurosurgery, Ruijin Hospital, Shanghai Jiao Tong University School of Medicine, Shanghai, China; 5 Institute of Neuroscience, Newcastle University, The Henry Wellcome Building, Newcastle upon Tyne, England, United Kingdom; Plymouth University, UNITED KINGDOM

## Abstract

Computational studies of the influence of different network parameters on the dynamic and topological network effects of brain stimulation can enhance our understanding of different outcomes between individuals. In this study, a brain stimulation session along with the subsequent post-stimulation brain activity is simulated for a period of one day using a network of modified Wilson-Cowan oscillators coupled according to diffusion imaging based structural connectivity. We use this computational model to examine how differences in the inter-region connectivity and the excitability of stimulated regions at the time of stimulation can affect post-stimulation behaviours. Our findings indicate that the initial inter-region connectivity can heavily affect the changes that stimulation induces in the connectivity of the network. Moreover, differences in the excitability of the stimulated regions seem to lead to different post-stimulation connectivity changes across the model network, including on the internal connectivity of non-stimulated regions.

## Introduction

Pharmaceutical drugs that can pass through the blood-brain-barrier lead to changes in the whole brain, which can result in severe side effects that have been documented in numerous clinical studies [1, 2]. Moreover, for many patients these traditional approaches do not work well in treating the symptoms of brain network disorders. Instead, targeted approaches that only *directly* affect a small number of brain regions have been proposed. These techniques range from localised opening of the blood-brain-barrier through focused ultrasound [3, 4], to invasive and non-invasive brain stimulation [5–8], and, when no alternative options are suitable, to surgical removal of brain tissue [9, 10]. The problem then is to choose the right set of target regions for individual patients to maximize treatment effects and to minimize side effects.

Parkinson’s disease and epilepsy are diseases where targeted approaches are already routinely used, when drug treatment is insufficient. For focal epilepsy, where medication is ineffective, resective surgery of the affected regions is often used as a treatment. However post-operative seizure remission is around 50–70% [11, 12]. The reoccurrence of seizures after surgery could be due to incomplete removal of the required target regions [13] or due to surgery causing remaining brain regions to become new starting points for seizures. For the latter option, it will be crucial to develop computer models of long-term effects of interventions.

The same challenge occurs for brain stimulation in epilepsy patients where no tissue is resected but where the stimulation of a target region, with reduction of epileptogenic activity in that region, could potentially cause other non-stimulated regions to become starting points for seizures. Targeted brain stimulation in epilepsy could include deep brain stimulation (DBS), optogenetic stimulation [14] (www.cando.ac.uk), and non-invasive techniques (transcranial current stimulation, TCS; transcranial magnetic stimulation, TMS). Moreover, techniques used in the treatment of other diseases, like the coordinated reset [15, 16] method (used for treatment of Parkinson’s) that aims to desynchronise neuronal populations (pathological synchronization being a major feature of epilepsy) could potentially be used in treating epilepsy. The effectiveness of the methods used varies [7] and when it comes to TCS–one of the non-invasive methods–there are of contradictory results concerning its efficacy for treating epilepsy [17–22].

One of the main concerns with TCS is whether the effects of stimulation would remain after the stimulation has ended [23]. Some studies have shown that the positive physiological effects of stimulation can outlast the stimulation session for a long period while others have shown diminishing effects after the stimulation session has ended. Specifically [24–26] have observed positive post-stimulation effects lasting for a period of 2, and more than 4 months respectively. On the other hand [27] observed anti-seizure effects for a period of 48 hours after stimulation but also a clinically significant reduction of those effects during a subsequent period of 4 weeks. To use computational models to assess the effect of brain stimulation, it is therefore, necessary to observe long-term changes.

At the moment, computational studies have only examined the short-term effects of TCS, i.e. during stimulation [28–32]. Two computational studies have used neural mass models [33, 34] to examine the immediate effects of stimulation on the activity of the stimulated areas. Notably, one study used modified Wilson-Cowan model to study effects a few minutes after anodal or cathodal stimulation [34]. The aforementioned studies did not account for plasticity in their models, and so did not investigate the effects of stimulation on brain connectivity, which is a proposed mechanism [35–37] by which TCS can affect brain activity in the long term. The only computational study to our knowledge that does examine the effects of neurostimulation on brain connectivity [38] focuses on DBS instead of TCS and examines Parkinson’s disease instead of epilepsy with the aim of identifying optimal stimulation locations.

In this study, we used a network of coupled modified Wilson-Cowan oscillators to examine how different aspects of the pre-stimulation brain connectivity affect the changes induced during and after a stimulation session. For this, connectivity data acquired from healthy and epileptic subjects was used to couple the nodes of the model network (to examine the effects of the inter-region connectivity and potential differences between the two groups) and two different versions of the stimulated nodes were examined (to see the effects of local excitability in the induced global changes), aiming to model healthy and epileptogenic brain regions respectively. Using this simplified model network, we simulated a single session of brain stimulation and the subsequent changes in connectivity for a period of 24 hours.

Our observations indicate long-term changes after the initial stimulation session in terms of both structural connectivity changes and changes in local and global network dynamics. Our analysis focused on connectivity changes as only such changes at the structural level can explain the behaviour of networks a long time after the initial stimulation and thus could potentially explain the variable final outcomes of treatment [39]. Our findings indicate that, simulated effects of brain stimulation differ when brain connectivity networks of healthy controls and epilepsy patients are used and moreover, stimulation leads to distinct long-term changes in the internal connectivity of non-stimulated regions, which appear hours after the end of the stimulation session.

## Methods

### Patient data

In order to initialise the connectivity of our models, we used data from 39 subjects, 19 of whom are suffering from left temporal lobe epilepsy. The subjects were selected from the dataset presented in [40, 41]. Written informed consent was obtained, signed by all participants, and conformed to local ethics requirements. The ethical review board of the medical faculty of Bonn gave IRB approval (032/08) and all experiments were performed in accordance with relevant guidelines and regulations. T1 weighted MRI scans and diffusion tensor imaging (DTI) data were obtained using a 3 Tesla scanner, a Siemens MAGNETOM TrioTim syngo (Erlangen, Germany). The T1 images were obtained using 1mm isovoxel, TR = 2500ms and TE = 3.5ms. The DTI data used 2mm isovoxel, TR = 10,000ms, TE = 91ms and 64 diffusion directions, b-factor 1000s mm−2 and 12 b0 images. In both caes FoV was 256mm.

To create the structural connectomes, FreeSurfer was used to obtain surface meshes of grey and white matter boundaries from the MRI data and to parcellate the brain into regions of interest (ROI) based on the Desikan atlas [42, 43]. This process identified 82 ROIs which spanned cortical and subcortical regions (Nucleus accumbens, Amygdala, Caudate, Hippocampus, Pallidum, Putamen and Thalamus). Streamline tractography was obtained from DTI images using the Fiber Assignment by Continuous Tracking (FACT) algorithm [44] through the Diffusion toolkit along with TrackVis [45]. First, we performed eddy-correction of the image by applying an affine transform of each diffusion volume to the b0 volume and rotating b-vectors using FSL toolbox (FSL, http://www.fmrib.ox.ac.uk/fsl/). After the diffusion tensor and its eigenvector was estimated for every voxel, we applied a deterministic tractography algorithm [44] initiating a single streamline from the centre of each voxel. Tracking was stopped when the angle change was too large (35 degree of angle threshold) or when tracking reached a voxel with a fractional anisotropy value of less than 0.2 [46].

**Fig 1:**
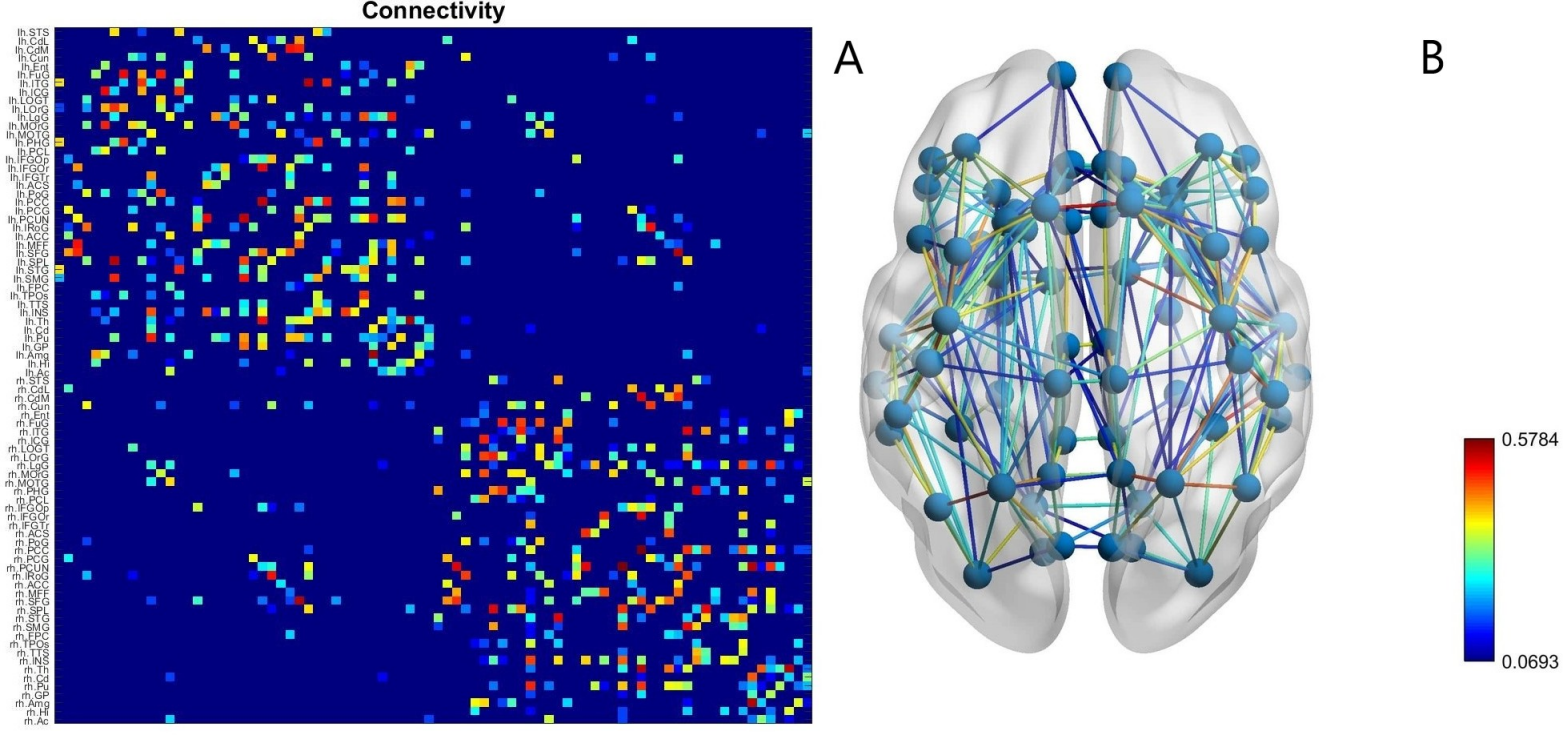
The connectivity matrix (A) obtained by the process described in the section Patient data for a healthy subject and (B) the network of nodes corresponding to that connectivity showing the positions of the brain regions represented by the network’s nodes. The strength of each connection (derived from the number of streamline counts between brain regions) is indicated by its colour.

The centre coordinates of each voxel were the start of a single streamline, the total number of streamlines never exceeded the number of seed voxels. The number of connecting streamlines were used to determine the connectivity matrix (S), as the streamline count has recently been confirmed to provide a realistic estimate of white matter pathway projection strength [47]. Distance matrices were also constructed using the mean fibre length of the streamlines connecting each pair of ROIs (Fig 1). The surface area of each ROI was found using FreeSurfer for cortical regions and for subcortical areas by computing the interface area to the white matter in T1 space [48].

### Modified Wilson-Cowan model

**Fig 2:**
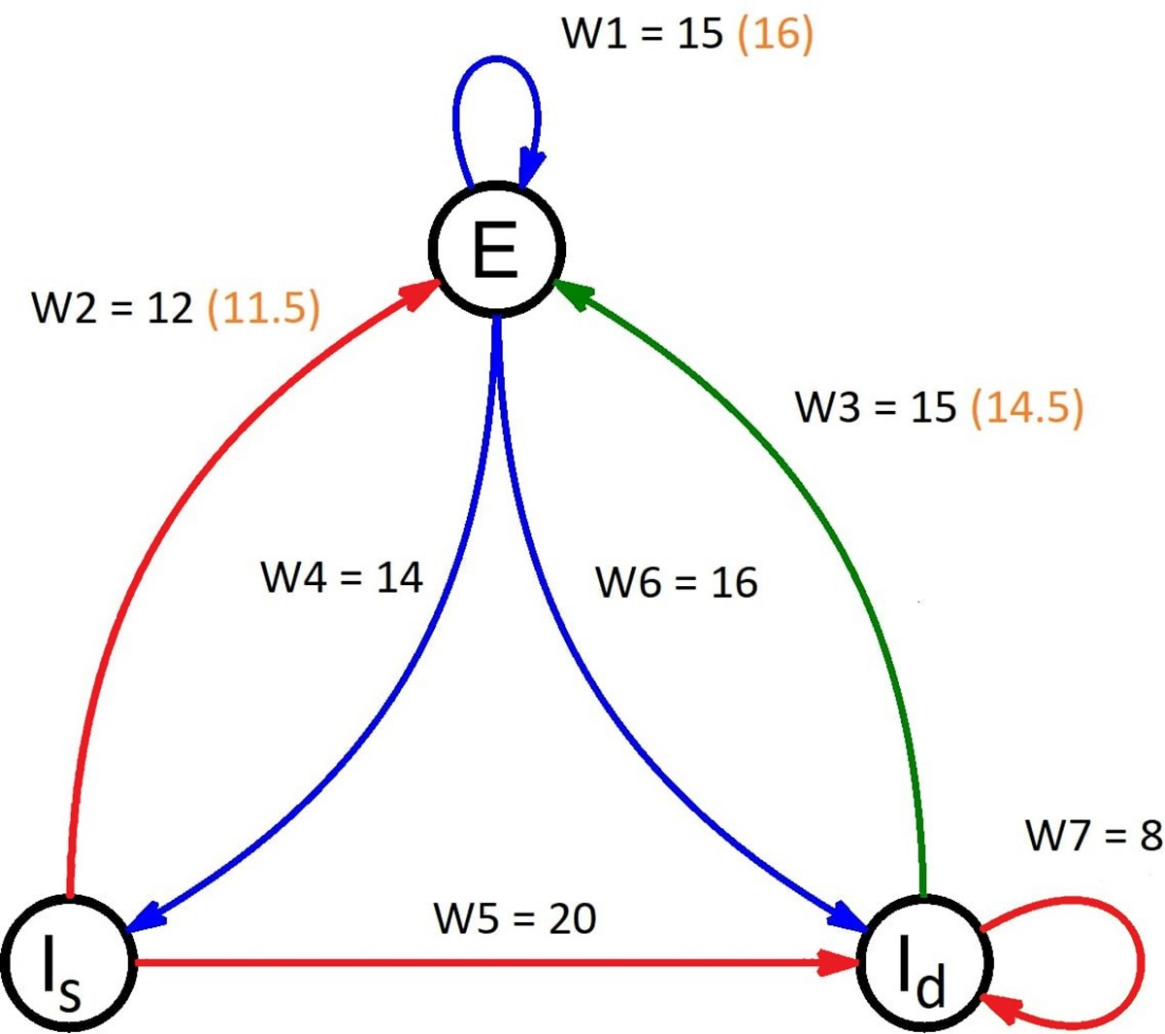
A diagram of a Wilson-Cowan node used in the model. The blue arrows indicate an excitatory connection while the red and green arrows indicate subtractive and divisive inhibitory connections respectively. The weights of each connection are indicated above every arrow. The numbers in the orange parentheses are the weight values that differ for the stimulated (epileptogenic) regions in the epileptic patients.

Our model consists of a network of 82 coupled modified Wilson-Cowan oscillators, each representing a single brain region. In order to include divisive inhibition into our model, each W-C node consists of one excitatory and two inhibitory populations (Fig 2). The first inhibitory population represents interneurons firing at the dendrites of the postsynaptic neurons (subtractive inhibition) and the second inhibitory population represents interneurons firing directly at the soma of the postsynaptic neurons, delivering divisive inhibition. For the implementation of the model we followed the methodology and notation of [49]. All the notations that we use for the description of the model are summarised in Table 1.

**Table 1:** Notation used in the text and interpretation.

Notation	Interpretation
*E*_*i*_(*t*)	Activity of the excitatory population of node i at time t
*Is*_*i*_(*t*)	Activity of the subtractive inhibitory population of node i at time t
*Id*_*i*_(*t*)	Activity of the divisive inhibitory population of node i at time t
$w_{k}^{\left(i\right)}$	Weight of the k-th connection of node i
*W*_*ij*_	Weight of the connection between nodes i and j
*del*_*ij*_	Time delay between nodes i and j
*P*_*e*_	External input of the excitatory population
*P*_*s*_	External input of the subtractive inhibitory population
*P*_*d*_	External input of the divisive inhibitory population
*F*_*e*_(*x*,*θ*,*a*)	Sigmoid function for the excitatory population
*F*_*i*_(*x*,*θ*,*a*)	Sigmoid function for the Inhibitory populations
*θ*	Variable of the sigmoid representing subtractive modulation
*a*	Variable of the sigmoid representing divisive modulation
*θ*_*e*_	Minimum displacement in case no subtractive inhibition is delivered to the excitatory population
*a*_*e*_	Maximum slope in case no divisive inhibition is delivered to the excitatory population
*θ*_*i*_	Minimum displacement in case no subtractive inhibition is delivered to the inhibitory populations
*a*_*i*_	Maximum slope in case no divisive inhibition is delivered to the inhibitory populations
*k*_*e*_	Constant for the excitatory population
*k*_*i*_	Constant for the inhibitory populations

Of course, the model described in [49] has been designed to simulate the connectivity of a cortical microcircuit and not the connectivity of sub-cortical regions. Still, a number of studies [50–52] have shown the presence of shunting inhibition (in addition to regular subtractive inhibition) in many of the subcortical areas we used in our study. Thus, we felt that the inclusion of both inhibitory populations in the nodes representing subcortical regions was justified.

According to this approach, the activity of each brain region is represented by a Wilson-Cowan model, governed by the following delayed differential equations (DDE’S):

$$\tau_{e}\frac{dE_{i}(t)}{dt}=-E_{i}(t)+(k_{e}-E_{i}(t))\cdot F_{e}(w_{1}^{\left(i\right)}{\cdot E}_{i}(t)+\sum_{j=1,j\neq i}^{82}W_{ji}\cdot E_{j}(t-del_{ij})+P_{e},\;w_{2}^{\left(i\right)}\cdot Is_{i}(t),\;w_{3}^{\left(i\right)}\cdot Id_{i}(t))$$ (1)

$$\tau_{i}\frac{dIs_{i}(t)}{dt}=-Is_{i}(t)+(k_{i}-Is_{i}(t))\cdot F_{i}(w_{4}^{\left(i\right)}{\cdot E}_{i}(t)+P_{s},0,0)$$ (2)

$$\tau_{i}\frac{dId_{i}(t)}{dt}=-Id_{i}(t)+(k_{i}-Id_{i}(t))\cdot F_{i}(w_{5}^{\left(i\right)}\cdot E_{i}(t)+P_{d},w_{6}^{\left(i\right)}\cdot Is_{i}(t)+w_{7}^{\left(i\right)}\cdot Id_{i}(t),\;0)$$ (3)

To account for the divisive inhibition a modified input-output function is required:

$$F_{j}(x,\theta ,a)=\frac{1}{1+\exp\;\left[-\frac{a_{j}}{1+a}(x-(\theta_{j}+\theta ))\right]}-\frac{1}{1+\exp\;\left[\frac{a_{j}\theta_{j}}{1+a}\right]}$$ (4)

For, *j*∈{*e*,*i*}, where *e* stands for excitatory and *i* stands for inhibitory. The inhibitory populations have the same input-output function and the same constants since they are assumed to respond to inputs in a similar way. However, the difference in the type of inhibition those neurons deliver to the excitatory population is due to their different targeting onto the postsynaptic neurons, that is, somatic vs dendritic.

The constant *k*_*j*_, *j*∈{*e*,*i*} is given by:

$$k_{j}=\lim_{x\to \infty }F_{j}(x,\theta ,a)=\frac{\exp\;\left[\frac{a_{j}\theta_{j}}{1+a}\right]}{1+\exp\;\left[\frac{a_{j}\theta_{j}}{1+a}\right]},\;j\in \{e,i\}$$ (5)

As is the case with the sigmoid function the constant *k*_*j*_ is the same for both inhibitory populations.

In our study, the constants of the sigmoid were set at *θ*_*e*_ = 4, *θ*_*i*_ = 3.7, *a*_*e*_ = 1.3, *a*_*i*_ = 2, following the values used at [49]. Moreover, the external inputs of the inhibitory populations were set to *P*_*s*_ = *P*_*d*_ = 1 while the input of the excitatory population was set to *P*_*e*_ = 2. Other values were considered for *P*_*e*_ ranging from 1.1 to 4 (the range where the system produces oscillations) with results similar to the ones presented here. Providing no input to the inhibitory populations (*P*_*s*_ = *P*_*d*_ = 0) results in a lack of long term stable oscillations and therefore we restricted the parameter value to *P*>0. A detailed description of all notation used is given in Table 1.

### Connectivity and plasticity

The weights *W*_*ij*_ between network nodes representing brain regions were initialized according to the brain anatomy of each patient using the data described in the section ‘‘Patient data”. Specifically, given the matrix *S* of the streamline counts for an individual subject we followed the original study [40] and initialised the connectivity matrix *M* as:

$$M_{ij}=\left\{ \begin{array}{c} 0.1\cdot \log\left(S_{ij}\right),S_{ij}>0\\ 0,\;S_{ij}=0 \end{array}\right.$$ (6)

This connectivity matrix was the only element of our study taken from biological data, everything else refers to simulations (and not experimental results) using the model described in this section.

The connectomes of healthy and epileptic patients did not show any apparent differences and due to the small dataset and very high dimensionality of the data (82x82 matrices) training (and testing) a classifier, to examine the possibility of distinguishing between the two groups at this level, was deemed unfeasible.

During the simulation, the weights were updated every 10 milliseconds by the following learning rule:

$$\Delta w_{ij}=c\cdot E_{i}(t-del_{ij})\cdot \left(E_{j}(t)-E_{j}(t-1)\right)$$ (7)

**Fig 3:**
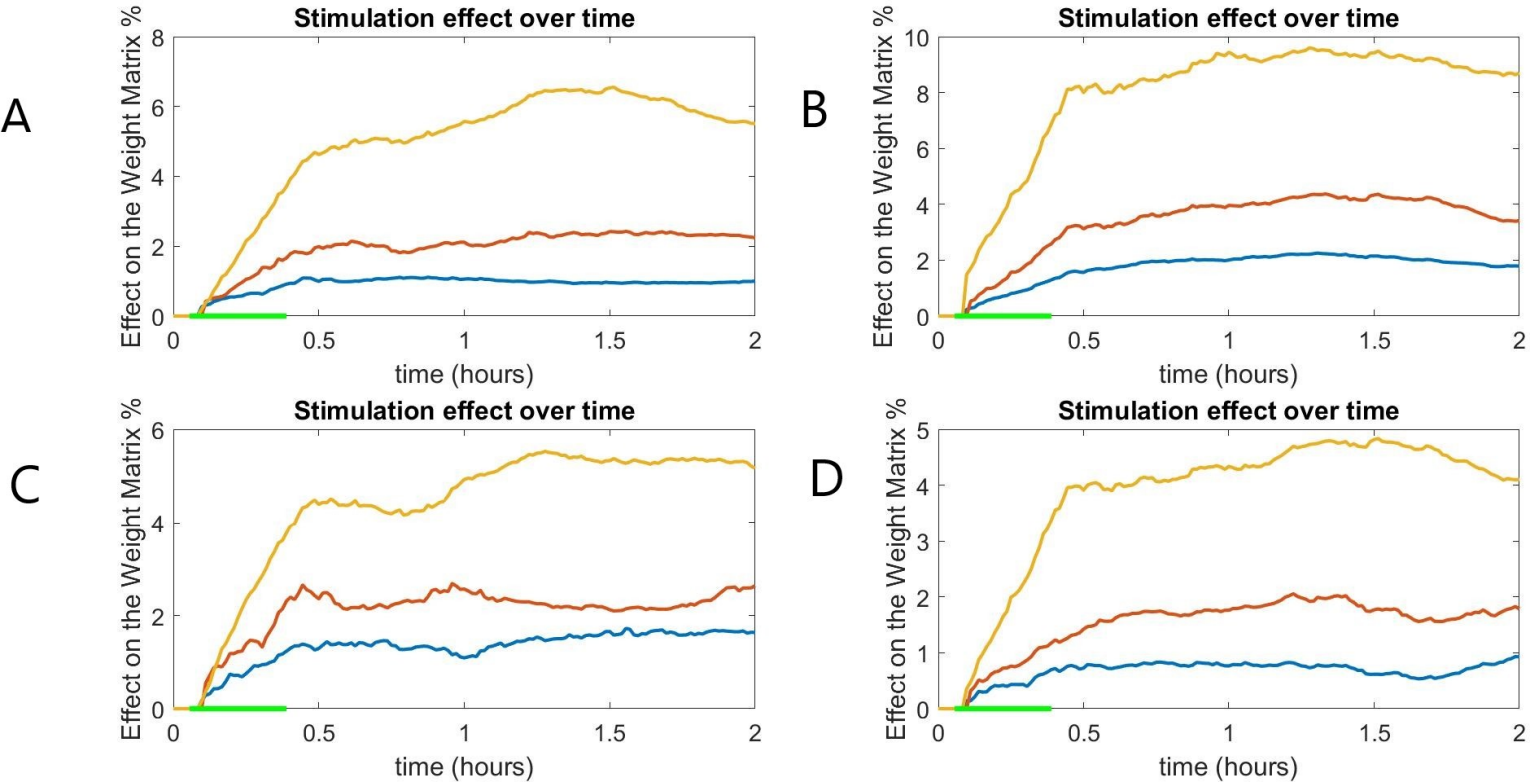
The global connectivity difference measures of two epileptic (A,B) and two healthy (C,D) subjects for different learning rates: c = 0.05 (blue), c = 0.1 (red) and c = 0.2 (yellow). The effect of stimulation on the global connectivity is different depending on the learning rate but the overall pattern remains similar. The green line at the x-axis indicates the period of stimulation.

We chose this simple rule in order to apply a simple form of Hebbian plasticity [53, 54] (if high activity in a pre-synaptic node is followed by an increase of activity in the post-synaptic node, the connection weight increases, otherwise it decreases) in neuron populations. The learning rate was set at c = 0.1. Other values were considered, and similar results were obtained with the only difference being the speed of weight change. Still, the pattern of activity remained the same for all the values we examined as can be seen in Fig 3.

The weight matrix was normalised after each update—to avoid runaway plasticity as indicated by the findings of [55, 56]—by the following rule:

$$W_{ij}\leftarrow \frac{W_{ij}}{\sum_{i=1}^{82}W_{ij}}$$ (8)

For the internal weights $w_{1}^{\left(i\right)},\ldots ,w_{7}^{\left(i\right)}$ of each node we used two different sets of initial values. The first set of values was chosen to represent the connectivity of a healthy brain region while the second set was chosen to represent an epileptogenic region. The values of the healthy region were decided after an extensive parameter search, starting at the values used by [49] and examining values between 8 and 21 (the range at which the system produces oscillations). The values we selected lead to high amplitude oscillations in all three populations during the first hours of the simulation. The amplitude of the oscillations gradually decreases and stabilizes after some hours. It must be noted that the final values were chosen to facilitate the dynamics of the system and may not correspond to the connectivity of a real biological system. Still, using different parameters usually resulted in oscillations of different amplitude and consequently slower stabilization periods, but as a general rule did not lead to radically different behaviour in the system.

After the values of the node representing a healthy region were established, the values of the nodes representing epileptogenic regions were derived by increasing the weights of excitatory connections and reducing the weights of the inhibitory connections. Those changes aimed at increasing the excitability of those nodes (increased excitatory and decreased inhibitory input) in order to simulate the dynamics associated with epilepsy. The difference in behaviour of the epileptogenic nodes was small but observable (oscillations of increased amplitude and occasional seizure-like activity when the input to their excitatory populations was increased), as with the original connection weights, choosing different values led to slightly different results (the more excitable a node is, the greater the effect of stimulation), but the main observations remained the same. The values chosen are presented in Fig 2.

The weights *w*_1_,*w*_2_,*w*_3_,*w*_4_,*w*_6_ were updated every 10 milliseconds according to a modified version of the rule we used for the external connections with subsequent normalization after every update.

$$\Delta w_{k}^{\left(i\right)}=c\cdot Pre(t)\cdot \left(Post(t)-Post(t-1)\right)$$ (9)

where *Pre*(*t*), *Post*(*t*) are the activities of the presynaptic and the postsynaptic populations, respectively. Several proposed mechanism of internal plasticity were considered, but due to the lack of a consensus about a general mechanism of inhibitory plasticity [53, 57]—especially in neural mass models—we chose to use this simple intuitive rule, similar to the rule we used for the external connections. The most commonly used learning rule for inhibitory plasticity, introduced in [58] could not be used in this model due to long term instability in the network’s dynamics.

For the normalization, we employed the same rule used for the global connectivity:

$$w_{k}^{\left(i\right)}\leftarrow \frac{w_{k}^{\left(i\right)}}{\sum_{k=1}^{7}w_{k}^{\left(i\right)}}$$ (10)

Since there has been little research on how inhibitory to inhibitory plasticity could be implemented in a neural mass model, the weights *w*_5_ and *w*_7_ were kept stable. The learning rate was set at c = 0.05.

Finally, the delays were initialized for each patient, as the length of the fibres connecting two brain regions divided by the speed of spike propagation. For the calculation of the delays we assumed that activity propagates with the same speed in all connecting fibres, which was set at 7 m/s, following the convention used at [59, 60]. To calculate the distance between regions, we selected the fibre trajectory length—which we calculated using deterministic tracking of diffusion tensor imaging data—instead of the Euclidian distance in order for the delays to be more biologically realistic.

### Stimulation

Each session of stimulation was modelled as a decrease of 50% (the stimulation is cathodal, due to better reported experimental results [21]) in the external input of three nodes representing the brain regions most commonly responsible for seizures in these patients (amygdala, hippocampus and parahippocampal gyrus), for a period of 30 minutes. Despite two of these brain regions being sub-cortical, the ability of transcranial stimulation to affect them has been demonstrated in past studies [61–63]. Stimulation in all cases started at t = 200s after the beginning of the simulation. This initial period was allowed for the oscillations of the system to stabilize before stimulation begins.

The choice of stimulation parameters was made in order for the model to correspond to a working protocol of TCS presented in [64]. Due to the computational constraints of such large simulations [65], we modelled only one session and an additional resting period of 24 hours.

### Implementation and analysis of results

Simulations were run for three distinct groups of subjects, according to the global connectivity data and model used:

1. Healthy subjects: The global connectivity data were derived from the healthy individuals and the simulation was performed using a model where no epileptogenic (particularly excitable) nodes are present.
2. Epilepsy patients: The global connectivity data were derived from individuals suffering from left temporal lobe epilepsy and the simulation was performed using a model where the stimulated nodes were modelled as epileptogenic (highly excitable)
3. Control subjects: The global connectivity data were derived from individuals suffering from left temporal lobe epilepsy but the simulation was performed using the “healthy” model, where the stimulated nodes are not distinct in terms of excitability from all other nodes.

After the initial choice of connectivity and model parameters, two simulations–with and without stimulation- run in parallel for a period of 24 hours with snapshots of the weight matrices taken every 50 seconds. The large system of DDE’s (246 equations) was solved by using Matlab’s dde23 delayed differential equation solver (The code for the model can be found in the following repository: https://github.com/MGiannakakis/Epilepsy_Simulation)

The effect of the stimulation on the connectivity at every time step was measured in the following ways:

1. The global effect of the stimulation on the connectivity of the brain was measured as the difference (%) of the connectivity matrices *M* = (*W*_*ij*_):
  $$D(t)=100\cdot \frac{\sum_{i,j=1}^{82}\left|{W\prime }_{ij}(t)-W_{ij}(t)\right|}{\sum_{i,j=1}^{82}\left|W_{ij}(t)\right|}$$ (11)
  where *W*′_*ij*_ is the weight between nodes *i* and *j* at time t after stimulation and *W*_*ij*_(*t*) is the weight between nodes *i* and *j* at time t without stimulation. This measure represents the effect stimulation has on the internode connections of the brain.
2. The effect of the stimulation on the internal connectivity of each node (local effect) was measured as the difference (%) of the internal weights in the stimulated and the non-stimulated versions:

$$d_{i}(t)=100\cdot \frac{\sum_{k=1}^{7}\left|w_{k}^{(i)\prime }(t)-w_{k}^{\left(i\right)}(t)\right|}{\sum_{k=1}^{7}\left|w_{k}^{\left(i\right)}(t)\right|}$$ (12)

where i = 1,…,82 the brain node, $w_{k}^{(i)\prime }(t)$ is the k-th weight of the i-th node at time t in the stimulated version and $w_{k}^{\left(i\right)}(t)$ is the i-th weight of the k-th node at time t in the non- stimulated version. These measures represent the effect of stimulation on the internal connectivity of each brain region.

### Connectivity measure

In order to study the effect of stimulation on the nodes that received no direct stimulation, we examined several connectivity metrics that could explain such an effect. One of those metrics is the Jaccard index. The Jaccard index of two nodes measures the similarity in connectivity (the common neighbours) and is defined as:

$$J(i,j)=\frac{\left|\Gamma (i)\cap \Gamma (j)\right|}{\left|\Gamma (i)\cup \Gamma (j)\right|}$$ (13)

Where *Γ*(*i*) is the set of nodes connected to node *i* and |*Γ*(*i*)∩*Γ*(*j*)|,|*Γ*(*i*)∪*Γ*(*j*)| are the number of elements in the sets *Γ*(*i*)∩*Γ*(*j*) and *Γ*(*i*)∪*Γ*(*j*) respectively.

In our study, we defined the Jaccard index of a secondary node *i* to be:

$$J(i)=\frac{1}{3}\cdot \left(J(p,i)+J(a,i)+J(h,i)\right)$$ (14)

Where *p*,*a*,*h* are the stimulated nodes.

## Results

Our results are organized in two sections. Firstly, we simulate the effect of stimulation on the overall connectivity of the network for each group of subjects. Secondly, we simulate the changes stimulation seems to induce in each node representing a brain region with emphasis at the stimulated nodes which represent the brain regions most often associated with seizure generation (amygdala, hippocampus and parahippocampal gyrus).

Statistical results will be presented for the rest of the paper as: X ± Y, where X is the mean and Y is the standard deviation of the referenced dataset. All the p-values were calculated using a two-tailed t-test.

### The network presents a larger global connectivity change at the end of the stimulation for epilepsy patient connectomes

**Fig 4:**
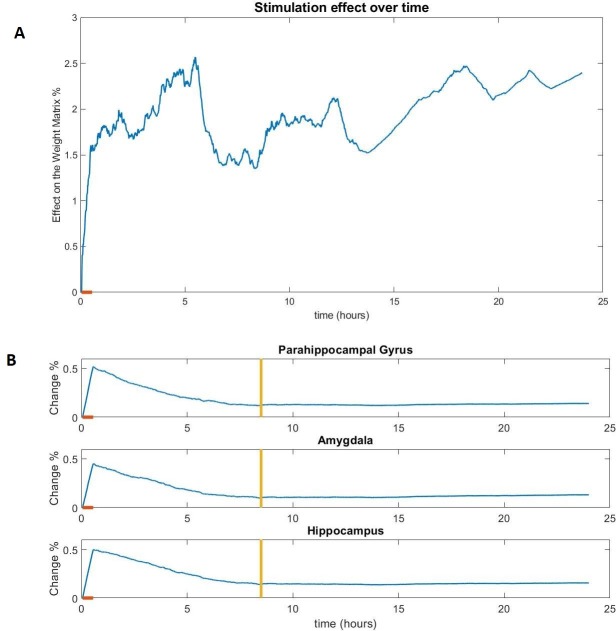
The effect of stimulation (difference from the non-stimulated version) on the global connectivity (A) and the connectivity of the stimulated nodes (B) of a healthy subject model. The orange line on the x-axis notes the duration of the stimulation session. The yellow vertical line notes the point of stabilization of the local measure d, consistently observed around t ≈ 8h for healthy and control subjects.

The effect of stimulation on the inter-node connections in our model follows a similar pattern in all subjects. Specifically, during the period of stimulation, the global effect measure *D*(*t*) increases steadily (Fig 4), reaching a local maximum at the end of stimulation (*t* = 2000 *s*). A first difference between the three groups can be observed at this point since the value of *D*(*t*) at the end of the stimulation session (30 min) is on average significantly (p-value < 0.0001) greater for the epileptic subjects (2.9730% ± 0.7301) than the healthy subjects (1.9671% ± 0.3261) and the control subjects (1.7609% ± 0.5290). The similarity of the healthy and control groups in contrast to the epileptic group suggests that the increased excitability of the stimulated nodes and not the initial global connectivity is the main driver of the changes of the global effect measure. Indeed, the global connectivity on its own seems to make the healthy subjects more excitable, since the values of *D*(*t*) were slightly higher for the healthy than the control group (although the difference was not statistically significant).

After the end of stimulation session, the global effect *D*(*t*) keeps fluctuating for the remainder of the simulation with a clear increasing trend in the majority of subjects. The rate of this increase varies greatly from subjects to subject and it was calculated as the rate *r* = *D*(*t*_0_)/*D*(*t*_1_), where *t*_0_ = 2000*s* is the end of the stimulation session and *t*_1_ = 24*h* the end of the simulation. For all subjects the value *d* varies greatly (0.5846% ± 0.2751) and we can also observe a small difference (statistically insignificant) between the values of healthy subjects (0.5358% ± 0.2128), the similar values of control subjects (0.5372% ± 0.1609) and the slightly greater values of epileptic subjects (0.6328% ± 0.2533) which is not statistically significant (S1 Fig). Thus, the differences between the groups are attributable to different effect of stimulation and not the post-stimulation change in connectivity.

Finally, in order to examine the extent to which the initial global connectivity determines the development of the global difference measure D, we measured the correlation between D in control and epileptic subjects initialised with the same connectivity data and found it to be higher (0.7747 ± 0.1102), than the average correlation between random pairs of control and epileptic subjects (0.6199 ± 0.3213). This, suggests that although the scale of change is mainly determined by the excitability of the stimulated nodes, the exact global connectivity does (at least partially) determine the development of the global effect measure.

### The inclusion of excitable nodes leads to a larger change in the local connectivity of the stimulated nodes during but not after stimulation

In the nodes that received direct stimulation (representing the amygdala, hippocampus and parahippocampal gyrus), the effect on the connectivity was most prominent during the period of stimulation, resulting in a constant increase of the local effect measure d_k_(*t*) in all three nodes. Thus, the local measure invariably reaches a global maximum at the end of the stimulation session (*t* = 2000 *s*). As with the global connectivity, the effect on the epileptic subjects is greater than the effect on the other two groups (p-value < 0.0001 for all three nodes). Specifically, the average effect for all three nodes on a healthy subject is 0.4746% ± 0.0509, in a control subject is 0.3853% ± 0.0427 and on an epileptic subject is 1.0794% ± 0.0264.

A difference from global connectivity is that in this case the difference between healthy and control subjects is clearly significant (p-value < 0.0001). This suggests that the brain connectivity of epileptic patients conditions the epileptogenic regions to be less excitable than in healthy individuals. Of course, the internal connectivity that makes these regions highly excitable masks this effect as we observed from the metrics of the epileptic group. Still, this finding seems to suggest that the inter-regional connectivity of epileptic patients tends to limit the excitability of epileptogenic regions.

After the end of the stimulation session, the local measure *d*_*k*_(*t*) changes similarly in the healthy/control groups but very differently in the epileptic group.

**Fig 5:**
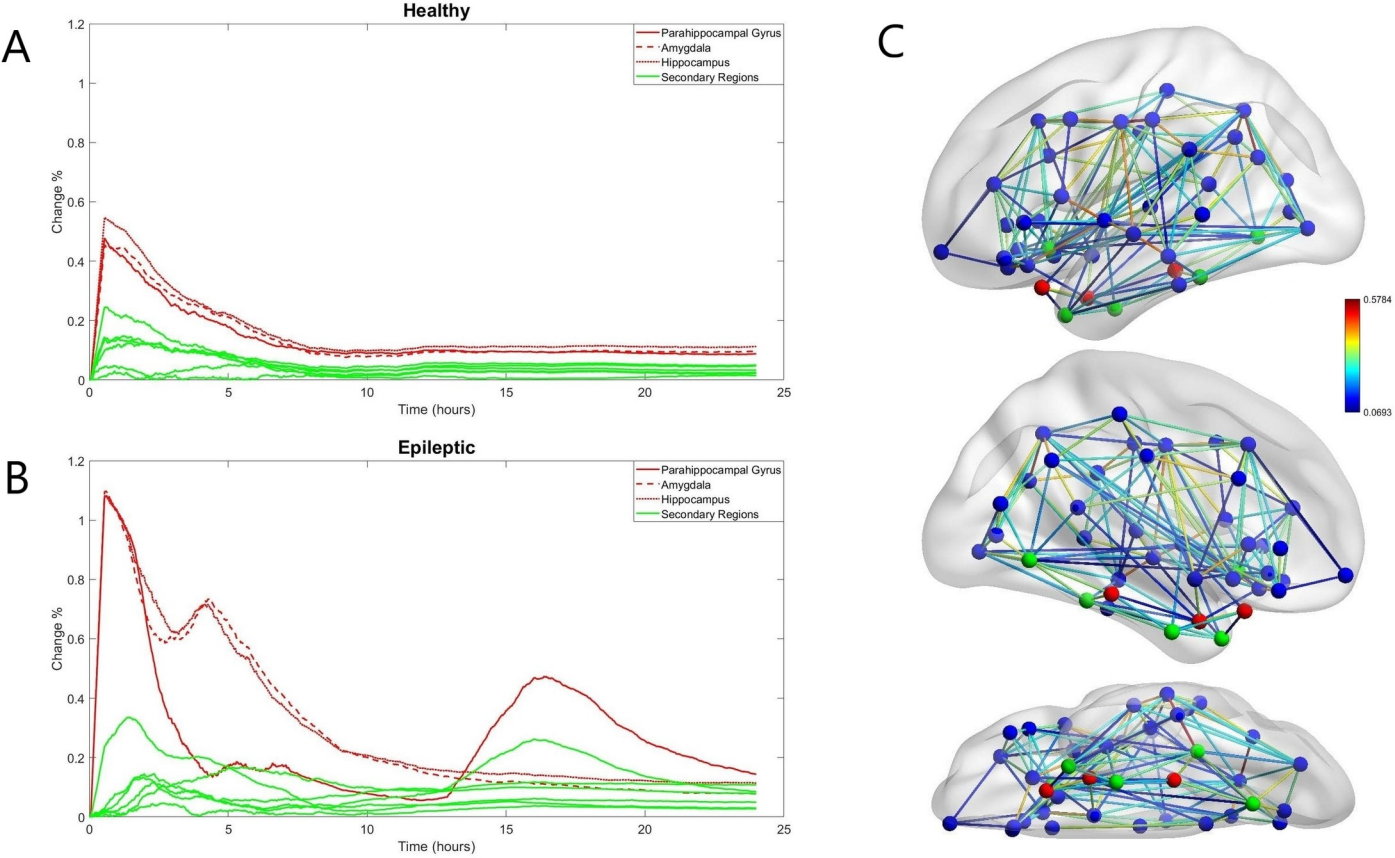
The local effect of the 3 stimulated nodes (in red) and 6 secondary nodes (in green) for a healthy subject (A) and an epilepsy patient (B). The location of these nodes in our model is shown in (C) for the brain connectivity of the epileptic subject.

In the healthy/control subjects, the end of the stimulation session (30 min) is followed by a slow decrease in the value of the local effect *d*_*k*_(*t*). Around 8 hours after the end of the stimulation session, the difference measure stabilizes at *d*_*k*_(*t*)≈0.1%, for all three nodes (Fig 4), for a representative subject. The local effect measure *d*_*k*_(*t*) of a node is considered to be stabilized at time *t* if the Coefficient of variation of the values of *d*_*k*_(*t*) for the 5 minutes prior to *t* is less than 0.3. After that point, there may be some small oscillation in the value of *d*_*k*_(*t*) but the change is minimal.There is much greater variation in the epileptic subjects, both between the nodes of the same subject as well as between equivalent nodes (representing the same brain region) of different subjects (Fig 5). Immediately after the end of the stimulation session and for a period lasting 5–6 hours, the local effect *d*_*k*_(*t*) is sharply (more than in the healthy/control subjects) decreasing for all 3 nodes. With the exception of two subjects where there is a short increasing period in the values of the amygdala and the hippocampus, *d*_*k*_(*t*) is strictly decreasing during this period for all three nodes of every subject. It should be noted that in almost all the epileptic subjects (18 out of 19), the connectivity of the node representing the parahippocampal gyrus is behaving differently than the connectivity of the nodes representing the other two stimulated regions. The local effect (measured by *d*_*k*_(*t*)) on the parahippocampal gyrus node is diminishing faster than the equivalent measures of the other two regions, reaching values close to zero at the end of this first period.

For the remainder of the simulation, each subject presents different behaviour and the various stimulated nodes also present differences in each subject. In 10 of the subjects the local effect on the node representing the parahippocampal gyrus remains at the low levels it reached in the end of the decrease period (1–5/6 hours) with some minimal increases. In the remaining 9 subjects the local effect on that node starts increasing at some point between 8–12 hours after the end of stimulation and continues to increase for the remainder of the simulation reaching values comparable with those of the other two regions. The nodes representing other two stimulated regions (amygdala and hippocampus) behave almost identically in each subject. After the end of the first period of decrease the local effect measures of these areas stabilize in 10 of the subjects and decrease very slowly in 6 of the subjects for the remainder of the stimulation. In the remaining 3 subjects, the local effect measure increases for a period of 1.5–2 hours until it reaches values much higher than those of the other subjects (*d*_*k*_(*t*)≈0.55), after that point the effect on those areas begins to slowly decrease.

At the end of the simulation, we can observe that the final values of d_k_(t) for the epileptic subjects (0.1412% ± 0.0882) are slightly greater than those of the healthy subjects (0.1165% ± 0.0275) which in turn are slightly greater than those of the control subjects (0.1037% ± 0.0400) in the nodes that received stimulation. Still that differences are not statistically significant. This implies that the initial difference between healthy/control and epileptic subject does not lead to a long-term difference in the stimulation effects.

### Some non-stimulated nodes show local connectivity changes after the end of the stimulation

The effects of stimulation can be seen not only on the internal connectivity of the nodes that are stimulated directly but also on the connectivity of other nodes that receive no direct stimulation (Fig 2).

Specifically, in all groups, the local effect *d*_*k*_(*t*) of several nodes starts increasing and reaches a peak shortly after the end of the stimulation session. It should be noted that the change in those nodes does not absolutely coincide with the stimulation session, rather it happens shortly afterwards, possibly due to the time delays. Moreover, unlike the directly stimulated nodes where a difference can be observed between the healthy/control and epileptic model groups, no such difference can be observed in the values of those secondary regions.

After this initial increase, the local effect on all secondary nodes usually decreases and seems to stabilize after a period of about 8 hours. For the majority of subjects (29 out of 39) the values that the difference measures have at this point will be very close to the values they will have at the end of the stimulation. In most cases, the final value of the effect measures for those nodes are very close to the values of the other non-stimulated nodes that were not affected by the stimulation, but in some cases the final values for some of these secondary nodes (especially the entorhinal cortex) are much closer to–and in some cases higher than—the values of the stimulated nodes. Interestingly, in some epileptic subjects (5 out of 19) the local effect measure of some secondary nodes began to suddenly increase hours after the stimulation session when they were apparently stabilised for some time. This unpredictable behaviour suggests than even in the very simplified model used for this study, the dynamics of plasticity are not easily predictable for a timescale of hours. That is an indication that long-term effects that cannot be predicted from the initial response to stimulation.

**Fig 6:**
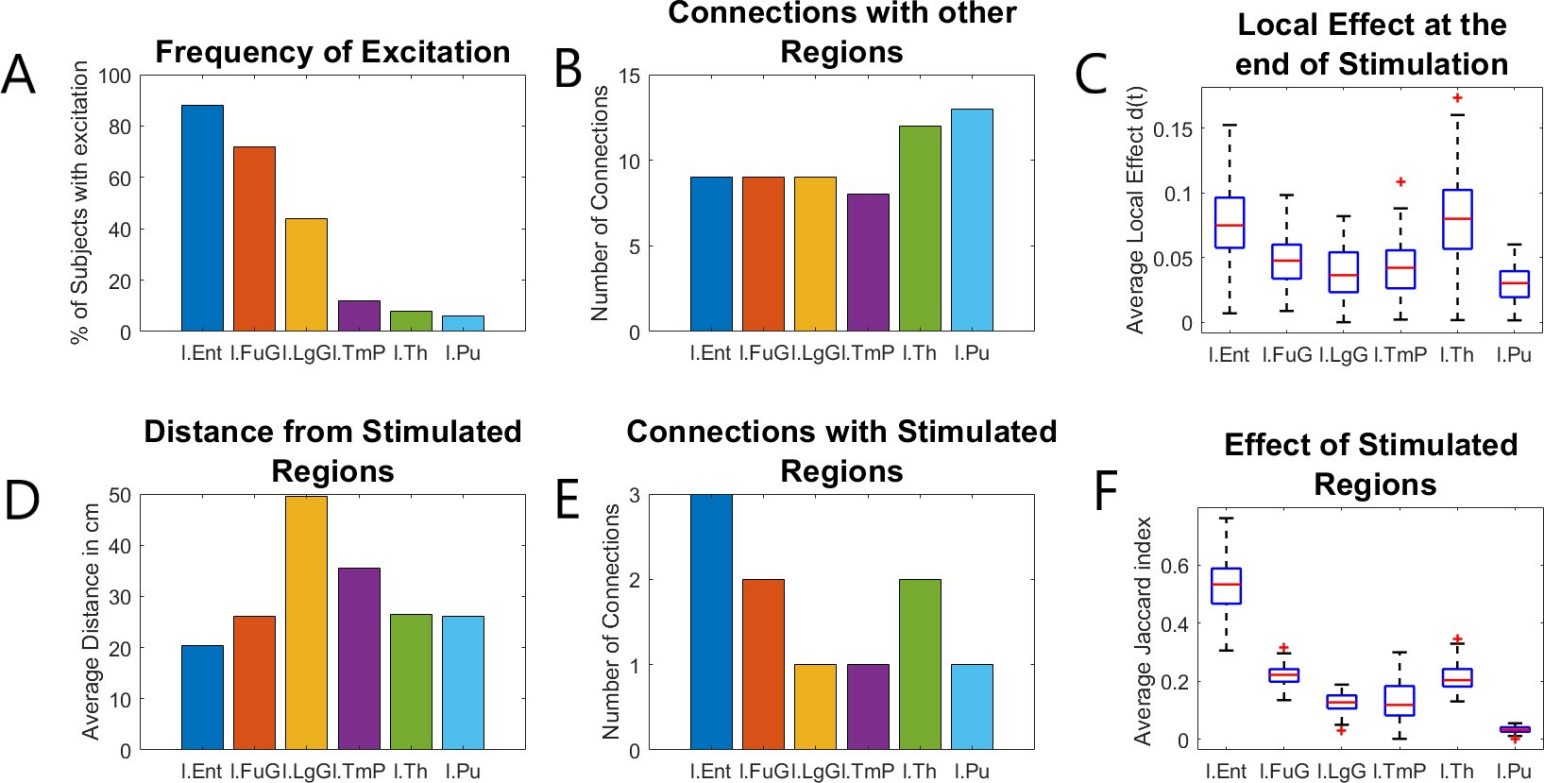
Metrics that could explain why secondary regions were affected. The most frequently excited secondary nodes, representing the Entorhinal Cortex, Fusiform Gyrus, Lingual Gyrus, Temporal Pole, Thalamus and Putamen of the left hemisphere, score higher in a variety of metrics that could explain why they are more affected than other nodes. Specifically, the frequency of Secondary excitation (A) is somewhat correlated with the amount of connections each node has with other regions (B) and especially the stimulated regions (E), as well as the average length of the connections with the stimulated regions (D). Moreover, the final value of the local effect d (C) as well as Jaccard index with the stimulated regions (F), seem to be strongly correlated with the frequency of secondary excitation.

Still, despite the fact that we do not know the exact cause of these post-stimulation changes, they seem to appear more frequently in some nodes than in others and several factors could explain why those nodes in particular were affected. Specifically, the brain regions that these nodes represent are characterized by increased connectivity with the stimulated regions as well as by a small Euclidian distance from the stimulated regions. Additionally, the effect the connections with the stimulated regions seemed to be greater than average (increased connection weights). Finally, the Jaccard index (common neighbours) of the affected nodes and the stimulated nodes was higher than in regions that were not affected. Moreover, the frequency of excitation among the six most commonly excited nodes (Fig 6) is correlated with the aforementioned metrics of the corresponding brain regions. For example, the node representing the entorhinal cortex that was affected in 17 of the subjects, scores higher in all the metrics (connectivity, Jaccard index, etc.) than the node representing the putamen which was excited in 2 of the subjects. A ranking of all the regions according to those metrics is given in Table 2. Moreover, the corresponding absolute values are presented in the supplementary information (S1 Table).

**Table 2:** A ranking of the nodes representing the regions of the left hemisphere according to several metrics that could explain secondary (non-stimulated) excitation. The table shows the ranking according to the frequency of those secondary effects, the local effect measure at the end of the simulation, the number of regions they are connected with and how many of these neighbouring regions are stimulated, the average effect of these stimulated regions (this was represented as the sum of the weights of the connections with stimulated regions divided with the sum of all weights) and their average Euclidian distance from the stimulated regions. The most commonly affected regions are shown in bold.

Node Index	Brain region with position corresponding to the network node	Value after stimulation	Value after 24h	Connectivity	Connections with stimulated regions	Effect of stimulated regions	Euclidian Distance from the stimulated regions	Jaccard Index
*1*	Banks of S.T.S	19	36	36	27	27	14	38
*2*	Caudal A.C	34	29	32	28	28	25	37
*3*	Caudal M.F	37	38	35	29	29	31	35
*4*	Cuneus	6	31	19	17	15	33	16
***5***	**Entorhinal C.**	**1**	**1**	**28**	**1**	**1**	**1**	**1**
***6***	**Fusiform G.**	**2**	**3**	**13**	**2**	**2**	**4**	**2**
*7*	Inferior Parietal	12	19	18	24	25	30	32
*8*	Inferior Temp.	14	11	27	12	10	7	6
*9*	Isthmus	7	13	20	4	4	15	7
*10*	Lateral Occipital	9	16	15	30	30	32	10
*11*	Lateral Orbit.	21	28	8	20	23	17	14
***12***	**Lingual gyrus**	**3**	**5**	**14**	**5**	**5**	**16**	**5**
*13*	Medial Orbit.	36	24	4	9	12	20	20
*14*	Middle Temp.	27	30	22	31	31	10	25
*15*	Paracentral	23	34	21	32	32	34	30
*16*	Pars Opercularis	30	33	26	33	33	18	29
*17*	Pars Orbitalis	33	26	33	21	19	26	28
*18*	Pars Triangular is	28	22	25	22	20	23	22
*19*	Pericalcarine	13	12	30	18	16	29	8
*20*	Postcentral	24	14	10	13	14	24	18
*21*	Posterior Cing.	35	21	17	34	34	19	26
*22*	Precentral	26	15	5	14	17	22	17
*23*	Precuneus	11	32	6	11	13	28	15
*24*	Rostral Ant. Cin.	29	23	31	35	35	27	36
*25*	Rostral M. Front.	31	20	12	23	21	35	27
*26*	Superior Frontal	20	35	2	19	24	36	23
*27*	Superior Parietal	15	17	9	16	18	37	19
*28*	Superior Temp.	17	25	11	36	36	9	24
*29*	Supramarginal	10	27	16	37	37	21	33
*30*	Frontal Pole	38	18	38	25	22	38	34
***31***	**Temporal Pole**	**4**	**4**	**23**	**6**	**6**	**12**	**3**
*32*	Trans. Temp.	18	37	37	38	38	8	31
*33*	Insula	25	9	1	8	11	6	11
***34***	**Thalamus**	**5**	**2**	**7**	**3**	**3**	**5**	**4**
*35*	Caudate	22	7	24	15	9	13	13
***36***	**Putamen**	**16**	**8**	**3**	**7**	**8**	**3**	**9**
*37*	Pallidum	8	6	29	10	7	2	12
*38*	Accumbens	32	10	3	26	26	11	21

We wondered whether these observed secondary effects may have clinical significance. The patients from whom the data originated had received resective surgery (not stimulation) of the seizure causing brain regions (amygdalohippocampectomy) and the outcome of these surgeries was known for a number of them (17 subjects). We found that the increased effect in the secondary nodes that was observed in the epileptic group was weakly correlated with a worse outcome of resective surgery: Epileptic subjects who presented a long-lasting effect on secondary nodes after stimulation within our model, i.e. higher values of the local effect measures compared with other non-stimulated nodes at the end of the simulation, were on average less likely (3.225 ± 1.220 on the ILAE classification scale) than those who did not present such effects (2.011 ± 1.110) to benefit from surgery (p-value = 0.0484, Cohen’s d = 1.042). Still, given the important differences between surgery and stimulation as well as the small sample size, it is very possible that this finding is not meaningful and further research with larger samples and experimental data from patients who received brain stimulation is required to evaluate any potential clinical application of this framework.

## Discussion

We investigated the effects of simulated cathodal TCS on the brain connectivity of healthy and epileptic subjects using a network of coupled Wilson-Cowan oscillators, which have been modified to include two inhibitory populations (for subtractive and divisive inhibition). Our results show that stimulation affects the simulated brain connectivity—a finding that has been confirmed by experimental studies [66]—as well as a significant difference between the effect stimulation has on different groups of subjects. Additionally, we have observed a great variability in the behaviour of our model after the end of the stimulation session, which can only be attributed to the differences in the initial patient-derived connectivity used for each simulation (the only factor differentiating the simulations of each group). Finally, we have observed that the effects of stimulation are not limited to the stimulated brain areas. In some patients the internal connectivity of a number of non-stimulated areas is affected by the stimulation of neighbouring areas and this seems to have a (weak but observable) correlation with a worse surgery outcome.

Our main observation is the different behaviour of our model under the different initialisations (healthy, epileptic and control groups). In all the cases we examined, the effect of stimulation both on the internal connectivity of the stimulated nodes as well as on the overall connectivity of the network was greater on the epileptic than the healthy and control subjects which behaved similarly. This difference, combined with the observation that the effect on the non-stimulated nodes was similar in all groups of subjects, suggests that the increased excitability of the epileptogenic nodes is responsible for the greater short-term effect of stimulation on the epileptic subjects.

Moreover, the significantly higher local effect of stimulation that was initially observed in the healthy subjects compared with the control subjects, suggests that there are indeed differences in the global connectivity of healthy and epileptic individuals and additionally indicates that the global connectivity of epileptic subjects tends to counter the epileptogenic effects of local connectivity. Finally, the long-term effects of stimulation on the internal connectivity were similar in all groups despite the initial differences, suggesting that the stimulation effect diminishes with different rates (faster in the more excitable regions) in each group.

Another finding is the great variation in the observed responses to stimulation among subjects of the same group. The extent to which the inter-regional connections change, the long-term preservation of the changes on the internal connections and the excitation of secondary nodes, differed a lot from subject to subject despite the fact that the initial connectivity matrix was the only factor differentiating the model used for each subject of a group. This fact suggests that the great variability in the effectiveness of stimulation may ultimately be caused by the differences in global brain connectivity among patients.

Finally, effects on the secondary nodes seem to appear without any prior indication, long after the end of the stimulation session. This observation, may indicate that effects of stimulation could appear long after the end of a session in brain regions where no stimulation was applied. In our study, we observed this phenomenon in almost 5 of the epileptic subjects within a period of 24 hours. We examined the possibility that these sudden changes in connectivity are due to computational errors in the simulation, but the fact that the nodes that present this sudden secondary excitation are almost always the ones that were affected immediately after stimulation (Table 1), suggests that this phenomenon is more likely attributable to the dynamics of the system and the underlying biological reality rather than to computational errors. Moreover, this phenomenon may be able to explain some of the unexpected long-term effects of TCS that appear in parts of the brain that were not stimulated. An example of this phenomenon is presented in [67], where seizures reoccur starting from a different brain region a month after an initially successful application of TCS.

## Limitations

Our study is far from conclusive for two main reasons. Firstly, the models we used are very rough approximations of the underlying biological reality and thus, the biological significance of our findings is far from certain. Special attention should be paid on the use of an unconventional learning rule as well as the fact that many of our constants were chosen to facilitate the simulation and thus, they may not represent the reality of biological systems. Also, local connectivity was initialised based on a previous model whereas measurements of fMRI allow for model parameters derived from subject-specific activity across brain regions [68].

Secondly, due to time limitations only one stimulation session was modelled with a subsequent resting period of 24 hours. Although our results do capture an abnormal behaviour (changes in secondary nodes), it is clear that given that in many of the studies discussed in the introduction the follow up period was ranging from several days to a little less than a year, our results may not represent the behaviour of biological systems for such long periods of time.

In addition to those two main issues, it should be noted that our dataset was quite small (19 patients and 20 controls) and thus the significance of our findings needs to be verified through larger datasets and experimental stimulation data. In particular, patient cohorts with brain stimulation data and simulation experiments of longer duration will be crucial to validate the predictive power of this model.

## Conclusion

This study uses computational methods to examine the long-term effects of TCS on the connectivity of the brain. Our findings indicate that even small differences in the internal connectivity—and thus the excitability—of the stimulated regions can radically change the way stimulation affects the brain. Moreover, the initial connectivity between brain regions also greatly affected the way each subject behaved post-stimulation. In addition, the effect stimulation has on non-stimulated brain regions seems to be a potential biomarker of long-term treatment outcome. Finally, sudden and seemingly unprovoked changes in the connectivity hours after the effects of stimulation could explain the unexpected effects of TCS that have been observed in the past.

## Supporting information

S1 Table Data for brain regions and the corresponding nodes in our simulation. (DOCX)

S1 Fig Boxplot of the global rates r for healthy and epileptic subjects. (TIF)
