# Supplementary material for: Computational modelling of the long-term effects of brain stimulation on the local and global structural connectivity of epileptic patients
Source: PLoS One. 2020 Feb 6;15(2):e0221380. doi: 10.1371/journal.pone.0221380 (PMC7004372; doi:10.1371/journal.pone.0221380)
Supplement: S1 Table — (DOCX) [file pone.0221380.s001.docx]

**Supplementary Information**

In S1 table we present the Absolute values from which the rankings presented in table 2 in the main text were derived:

**S1 Table. Data for brain regions and the corresponding nodes in our simulation.**

| Node Index | Brain region with position corresponding to the network node | Frequency of excitation (number of subjects) | Average value of  $\boldsymbol{d}_{\boldsymbol{k}}\boldsymbol{(t)}$ after stimulation | Average value of  $\boldsymbol{d}_{\boldsymbol{k}}\boldsymbol{(t)}$ after 24H | Connections with other regions / connections with stimulated regions | Average effect of stimulated regions | Euclidian Distance from the stimulated regions | Jaccard Index |
| --- | --- | --- | --- | --- | --- | --- | --- | --- |
| 1 | Banks of S.T.S | 0 | 0.021534±0.00012187 | 0.021626±0.00015088 | 5/0 | 0±0 | 42.8337 | 0.0027458 |
| 2 | Caudal A.C | 0 | 0.015924±8.3243e-05 | 0.022633±0.00020389 | 5/0 | 0±0 | 59.0435 | 0.0048979 |
| 3 | Caudal M.F | 0 | 0.014933±8.3924e-05 | 0.020929±0.0001628 | 5/0 | 0±0 | 66.4518 | 0.024108 |
| 4 | Cuneus | 0 | 0.035738±0.00035482 | 0.022245±0.00026765 | 8/0 | 0.012548±0.001104 | 69.5118 | 0.083075 |
| 5 | Entorhinal C. | 17 | 0.17878±0.0055843 | 0.076634±0.0013457 | 6/3 | 0.52332±0.0075533 | 20.3991 | 0.2631 |
| 6 | Fusiform G. | 14 | 0.098172±0.0032875 | 0.04703±0.00051646 | 9/2 | 0.22403±0.001619 | 26.185 | 0.24439 |
| 7 | Inferior Parietal | 0 | 0.024792±0.00020002 | 0.025132±0.00018663 | 8/0 | 0.0014978±8.749e-05 | 65.0172 | 0.03321 |
| 8 | Inferior Temp. | 0 | 0.024179±0.00034635 | 0.026987±0.0002009 | 7/0 | 0.024369±0.0021221 | 30.7728 | 0.13158 |
| 9 | Isthmus | 0 | 0.030982±0.0010198 | 0.026703±0.00022944 | 8/1 | 0.14377±0.0074791 | 43.2005 | 0.12948 |
| 10 | Lateral Occipital | 0 | 0.026634±0.00024186 | 0.02589±0.00023544 | 9/0 | 0±0 | 67.5363 | 0.10641 |
| 11 | Lateral Orbit. | 0 | 0.020148±0.0001096 | 0.022888±0.00020813 | 12/0 | 0.0024321±9.7431e-05 | 49.7472 | 0.096052 |
| 12 | Lingual gyrus | 8 | 0.091978±0.0020088 | 0.035414±0.00053991 | 9/3 | 0.12834±0.00095699 | 49.52 | 0.14531 |
| 13 | Medial Orbit. | 0 | 0.015417±0.0001408 | 0.023591±0.0001722 | 12/0 | 0.020188±0.00065506 | 53.6211 | 0.073411 |
| 14 | Middle Temp. | 0 | 0.01852±0.00013691 | 0.022633±0.00014591 | 8/0 | 0±0 | 33.894 | 0.049503 |
| 15 | Paracentral | 0 | 0.019889±0.00014633 | 0.022094±0.00019844 | 8/0 | 0±0 | 69.5889 | 0.039253 |
| 16 | Pars Opercularis | 0 | 0.017129±0.0001072 | 0.022104±0.00022522 | 7/0 | 0±0 | 50.0027 | 0.042008 |
| 17 | Pars Orbitalis | 0 | 0.016101±0.00012744 | 0.023287±0.00021246 | 5/0 | 0.0064314±0.00058303 | 59.7897 | 0.043532 |
| 18 | Pars Triangular is | 0 | 0.017657±0.00015257 | 0.024005±0.00024884 | 7/0 | 0.0051365±0.00035499 | 57.9594 | 0.059709 |
| 19 | Pericalcarine | 0 | 0.024496±0.0002976 | 0.026769±0.00035365 | 6/0 | 0.011305±0.0011263 | 62.8524 | 0.11367 |
| 20 | Postcentral | 0 | 0.019224±0.00022521 | 0.026188±0.00028871 | 11/0 | 0.01272±0.00055345 | 58.5126 | 0.077367 |
| 21 | Posterior Cing. | 0 | 0.015631±0.00010508 | 0.024321±0.00017182 | 8/0 | 0±0 | 52.9769 | 0.045864 |
| 22 | Precentral | 0 | 0.018884±0.00011873 | 0.026043±0.00054976 | 12/0 | 0.0080902±0.00027094 | 57.5903 | 0.07882 |
| 23 | Precuneus | 0 | 0.025677±0.00026157 | 0.022154±0.00020438 | 12/0 | 0.016533±0.00083557 | 62.9237 | 0.093859 |
| 24 | Rostral Ant. Cin. | 0 | 0.017487±0.00013064 | 0.023628±0.00014327 | 6/0 | 0±0 | 60.3329 | 0.018522 |
| 25 | Rostral M. Front. | 0 | 0.017085±0.00017179 | 0.024723±0.0002151 | 10/0 | 0.0032211±0.0001498 | 71.3106 | 0.044089 |
| 26 | Superior Frontal | 0 | 0.020288±0.00019581 | 0.022026±0.00012282 | 14/0 | 0.0021933±5.8373e-05 | 72.5067 | 0.055156 |
| 27 | Superior Parieta | 0 | 0.023797±0.00026766 | 0.025803±0.00020515 | 11/0 | 0.0074278±0.00033246 | 72.6635 | 0.07367 |
| 28 | Superior Temp. | 0 | 0.022671±0.00021075 | 0.023429±0.00021781 | 10/0 | 0±0 | 32.4339 | 0.05294 |
| 29 | Supramarginal | 0 | 0.026317±0.00026955 | 0.022938±0.00019825 | 9/0 | 0±0 | 55.6969 | 0.027949 |
| 30 | Frontal Pole | 0 | 0.01398±5.5621e-05 | 0.025343±0.00038338 | 3/0 | 0.0027784±0.00030105 | 84.1793 | 0.025239 |
| 31 | Temporal Pole | 3 | 0.053685±0.0013795 | 0.04259±0.00067892 | 8/1 | 0.11288±0.0058537 | 35.4618 | 0.20885 |
| 32 | Trans. Temp. | 0 | 0.021575±0.00014418 | 0.021255±0.00024119 | 4/0 | 0±0 | 30.8448 | 0.033846 |
| 33 | Insula | 0 | 0.01902±0.00019071 | 0.027938±0.00037618 | 12/2 | 0.022425±0.0006397 | 27.7651 | 0.10456 |
| 34 | Thalamus | 3 | 0.039393±0.00098343 | 0.071422±0.0028387 | 13/1 | 0.208±0.0023388 | 26.4036 | 0.14671 |
| 35 | Caudate | 0 | 0.020132±0.0001803 | 0.034276±0.00058299 | 7/0 | 0.025139±0.0030446 | 38.6088 | 0.097572 |
| 36 | Putamen | 2 | 0.02346±0.00048434 | 0.028582±0.00022917 | 13/1 | 0.035708±0.0010945 | 26.1245 | 0.10708 |
| 37 | Pallidum | 0 | 0.02873±0.00041973 | 0.035332±0.0010208 | 6/0 | 0.060426±0.0084081 | 22.947 | 0.098297 |
| 38 | Accumbens | 0 | 0.0165±0.00014437 | 0.027843±0.00029665 | 5/0 | 0.0014644±8.3632e-05 | 34.1467 | 0.063867 |

**S1 Fig. Boxplot of the global rates r for healthy and epileptic subjects.**
