# Supplementary figures and images for: Computational modelling of the long-term effects of brain stimulation on the local and global structural connectivity of epileptic patients
Source: PLoS One. 2020 Feb 6;15(2):e0221380. doi: 10.1371/journal.pone.0221380 (PMC7004372; doi:10.1371/journal.pone.0221380)

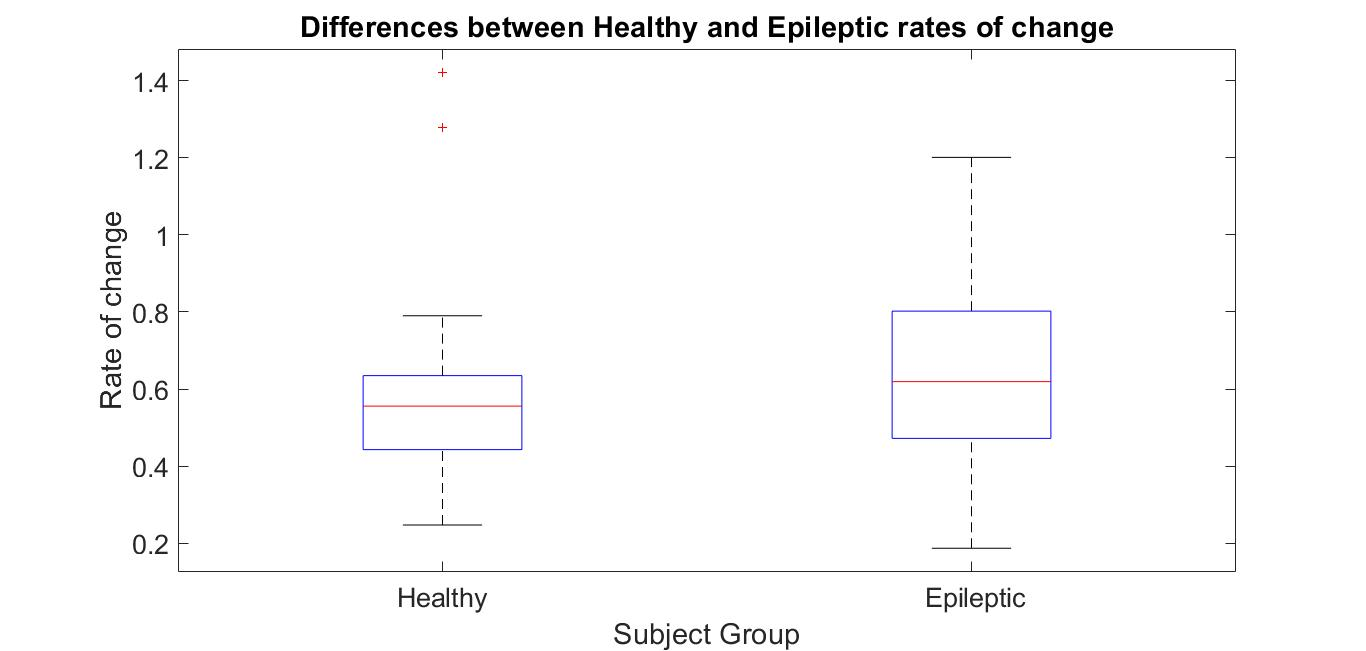

Supplement: S1 Fig — (TIF) [file pone.0221380.s002.tif]
